# A rare case of bilateral double incisors; Early orthodontic management

**DOI:** 10.1002/ccr3.4265

**Published:** 2021-06-23

**Authors:** Soghra Yassaei, Mohadeseh Sharifi, Zahra Ebrahiminik

**Affiliations:** ^1^ Department of Orthodontics Faculty of Dentistry ShahidSadoughi University of Medical Sciences Yazd Iran; ^2^ Department of Orthodontics Dental School of Aja University of Medical Sciences Tehran Iran

**Keywords:** double teeth, orthodontics, midpalatal suture

## Abstract

A safe and suitable approach in the treatment of unusual malformed anterior maxillary teeth would be to accept a birooted fused incisor as two central teeth by moving it across the midpalatal suture and improve the frontal esthetics.

## Graphical abstract

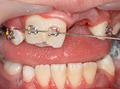

## 1 INTRODUCTION

Early improvement of esthetic smile is critical in patients with severely malformed front teeth particularly when it affects the patient's confidence. As illustrated, correction of midline by moving a fused central incisor across the midpalatal suture was successfully performed to make an acceptable esthetic smile.

Double tooth is a general word commonly used to describe either fusion or gemination.^1^ The literature showed prevalence estimates for bilateral double teeth ranging from 0.01% to 0.04% in the primary dentition and 0.05% in the permanent dentition.^1^, ^2^ Differential diagnosis between these two anomalies can be challenging; however, tooth count is always the first step.^3^ In germination, tooth count is normal, while in fusion, the number of teeth is one fewer unless it happens between a supernumerary tooth and a normal one.^2^, ^4^

These anomalies could lead to higher caries potential, malocclusion, changes in the dental arch shape, periodontal disease, hyper‐ /hypodontia, and poorer esthetics. Treatment options include restorative treatment (35%), hemisection (33%), extraction (15%), and no intervention (17%) based on Smail‐Faugeron's report.^5^ Orthodontic treatment has been reported to be a main or alternative option in 57% of the cases with double teeth anomaly.^5^, ^6^

Animal studies^7^ and case reports^8^, ^9^, ^10^ have shown that movement of the teeth across the midpalatal suture (MPS) is biologically possible; however, it always offers a unique challenge for the orthodontists. The main considerations are root resorption and frenum inflammations.^9^ Jason pair in 2011 represented a case of bilateral gemination in which they extracted the right incisor and moved the left one 3 mm across the suture to correct the midline.^10^ In their case, the geminated tooth had smaller widths compared to a normal one.^10^ Garib (2012) also reported a case that the right incisor moved through the suture to replace the absent contralateral tooth.^8^ In both cases, frenectomy has been done to decrease the inflammation of stretched frenum, and also to decrease the chance of relapse. However, no major root resorption was reported by any of those studies. In this study, a rare case of moving a birooted fused central incisor across the MPS is presented.

## 2 CASE PRESENTATION

A 10‐year‐old male patient presented to the orthodontic department of Shahid Sadoughi University of Medical Sciences with the main complaint of enlarged front teeth. The patient was the first of 3 siblings of parents with no history of consanguinity. The patient appeared normal and healthy with no reported history of orofacial trauma.

### 2.1 Diagnosis

**FIGURE 1:**
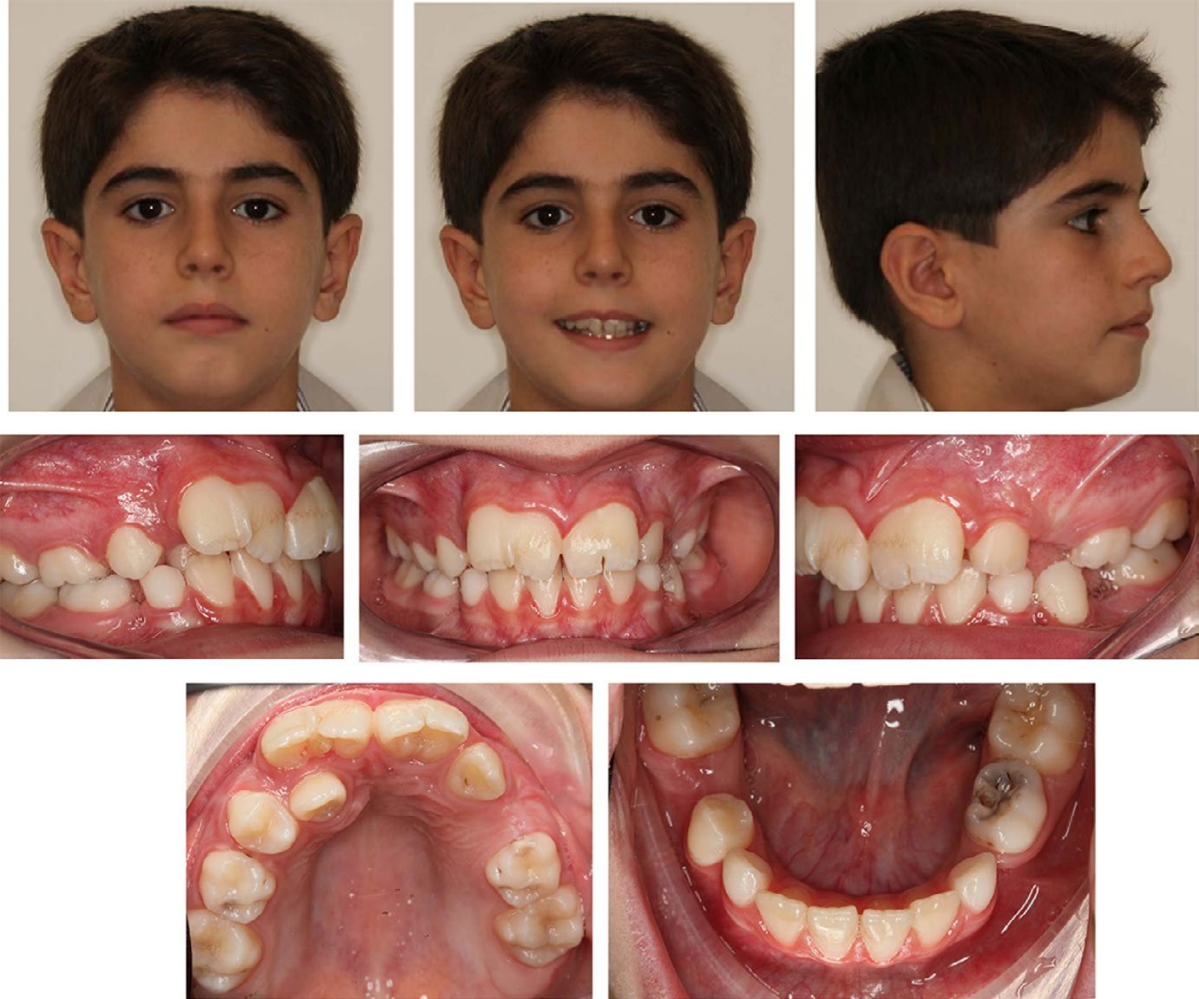
Pretreatment extraoral and intraoral photographs

On examination, the maxillary right (#11) and left (#21) central incisors had increased mesiodistal widths with slight notching presented in the incisal region extending through the labial cervical third. Both incisors were within the arch form with no evidence of any caries. The remaining teeth were of normal size and shape, and the total number of teeth was normal (Figure 1).

**FIGURE 2:**
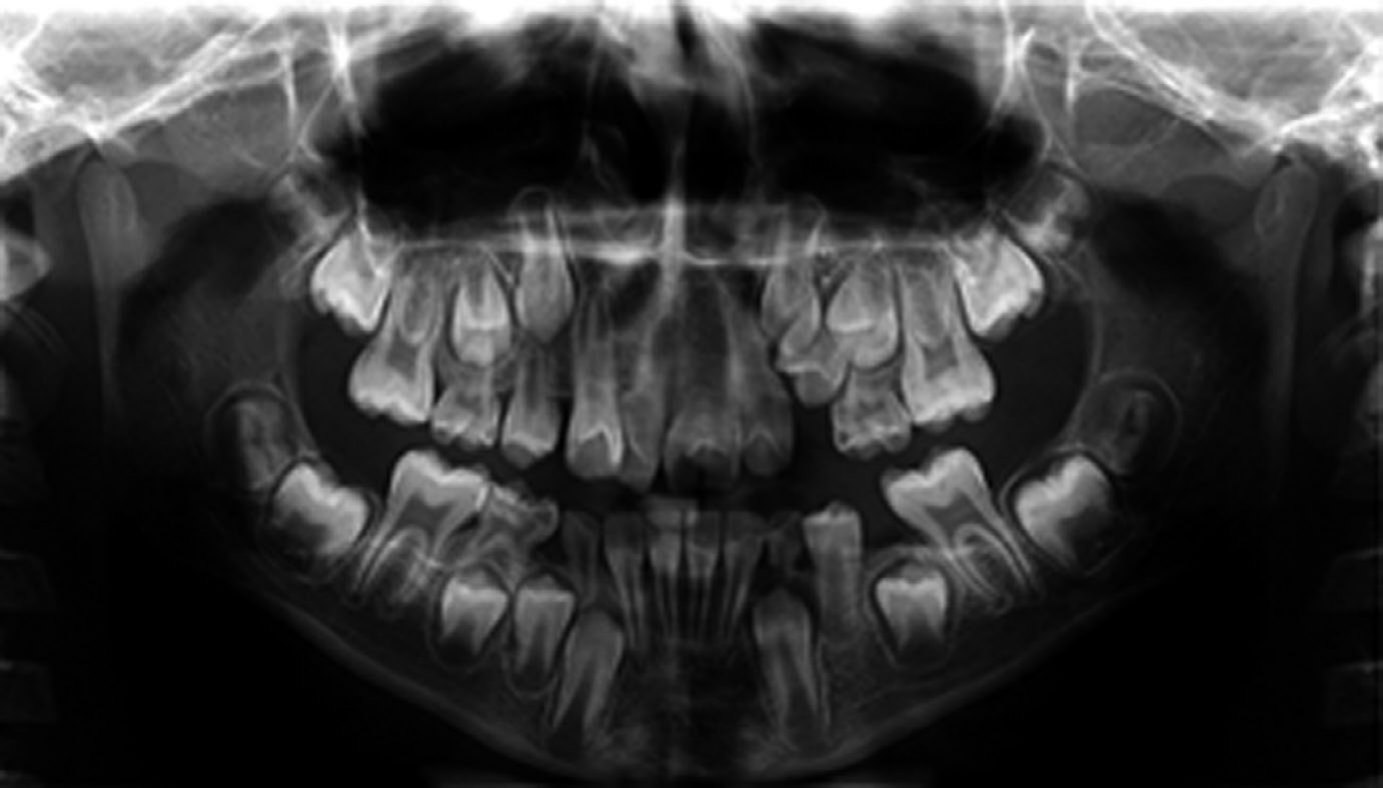
Pretreatment panoramic radiograph

Radiographic evaluations (Figure 2) showed that #21 had a single root and a common pulp chamber, while #11 had two distinct diverging roots and their pulp chambers were individualized. The diagnosis was made to be bilateral double teeth which #21 was an incomplete gemination and #11 was a fusion between the incisor and a supernumerary tooth.

**FIGURE 3:**
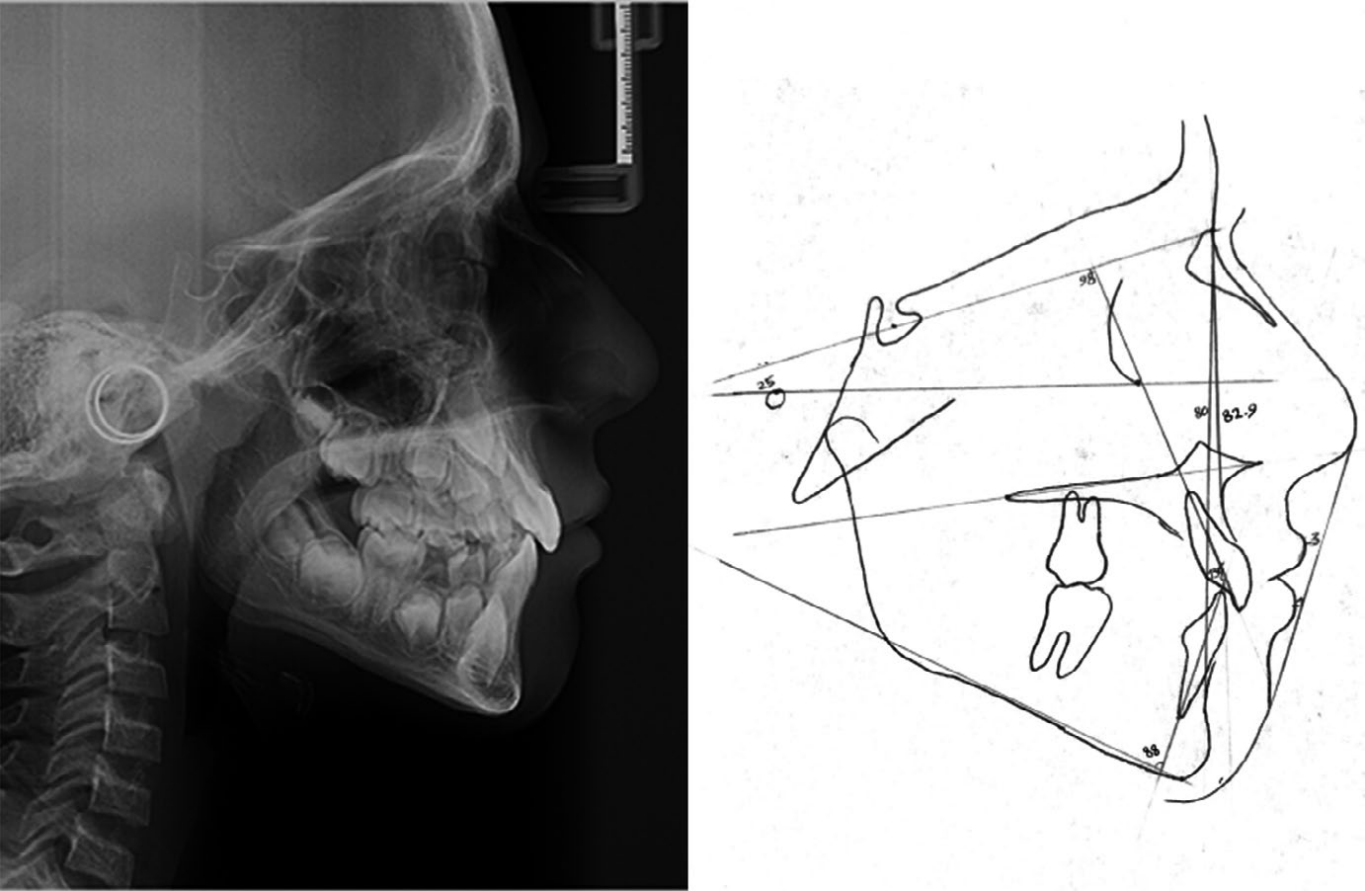
Pretreatment lateral cephalogram and tracing

**FIGURE 4:**
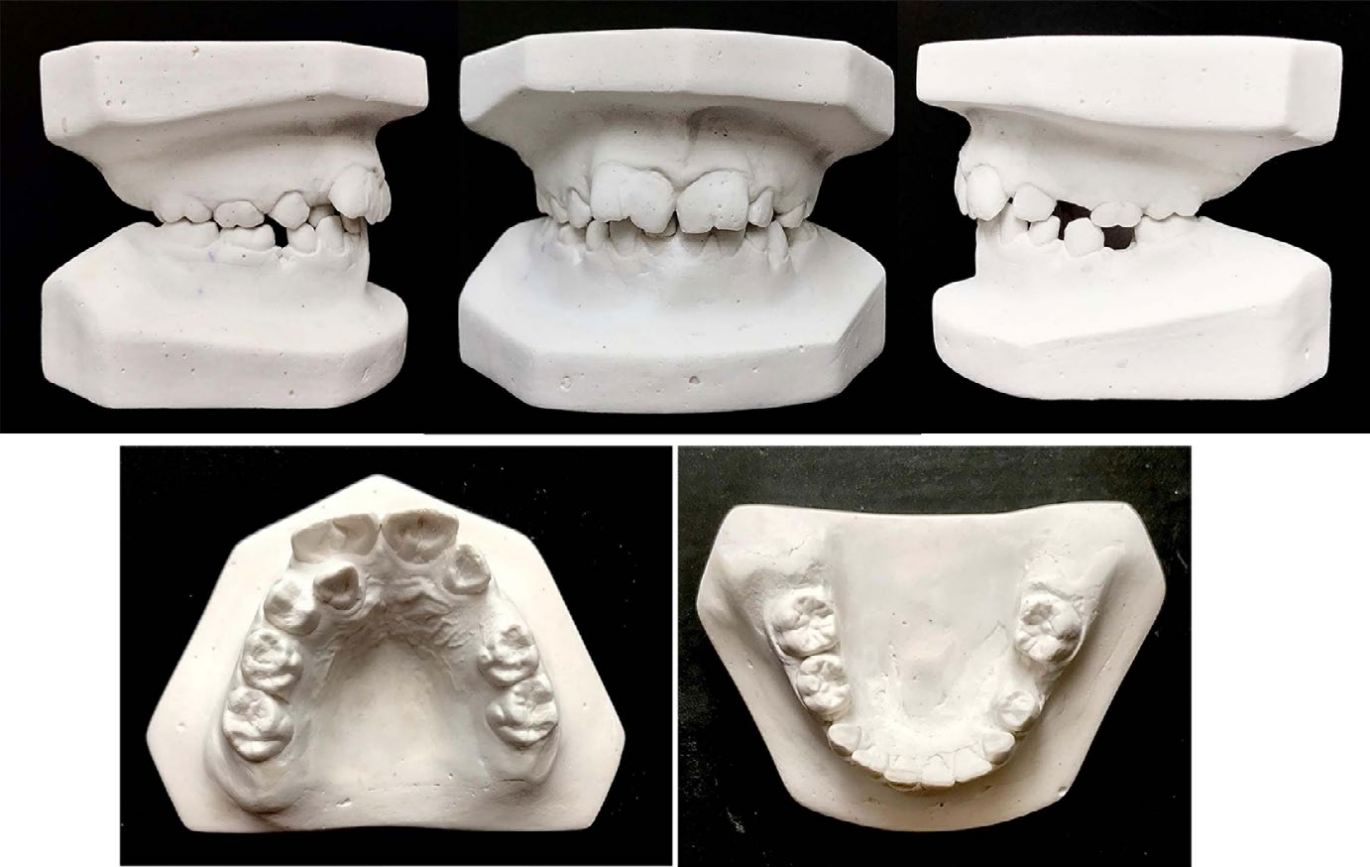
Pretreatment dental casts

Lateral cephalogram analysis (Figure 3) showed skeletal class I and a slight tendency to the vertical growth pattern. Dental cast (Figure 4) analysis showed severe space deficiency in both arches (12 mm in the upper arch and 11 mm in the lower arch).

### 2.2 Treatment objectives

Treatment objectives were to increase the patient's confidence by restoring smile esthetic, also to establish a class I occlusion, an ideal overjet/overbite, and to maintain the facial profile proportions.

The treatment plan was to reduce the front dental mass by extraction of #21 and mesializing #11 to correct the midline. Also comprehensive orthodontic treatment with four first premolar extractions was anticipated for relieving the severe crowding.

### 2.3 Treatment alternatives

Alternative treatment plans include extraction of two anterior double incisors and implant placement, or reduction of the incisor's widths by splitting/hemisection and improvement of the esthetic by ceramic crowns. These options would be opened only after the patient's skeletal maturation. Moreover, surgical, endodontic, and prosthetic procedures were needed for those options. The patient preferred the less aggressive procedure; therefore, ext of #21 and mesialization of #11 was the chosen plan. The patient and his family approved the publication of the treatment records.

### 2.4 Treatment progress

**FIGURE 5:**
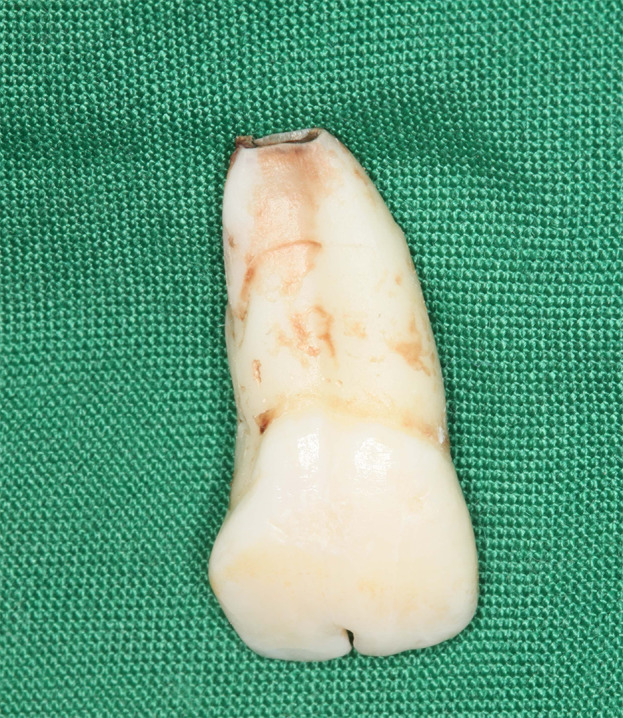
The extracted #21 tooth mass had one apex and one single, enlarged pulp canal

Due to the young age of the patient, emphasis on oral hygiene instruction was always a priority of the visiting sessions. In the beginning, ext of #21 was ordered (Figure 5) and it was planned to consider #11 as two central incisors (readily existing in the form of a single macrodontic, fused tooth).

One week after the extraction of #21, bracket bonding of maxillary teeth (including the primary canines and molars) was performed (0.028 × 0.022‐in slot size bracket, discovery^®^ smart, Dentaurum, Germany).

**FIGURE 6:**
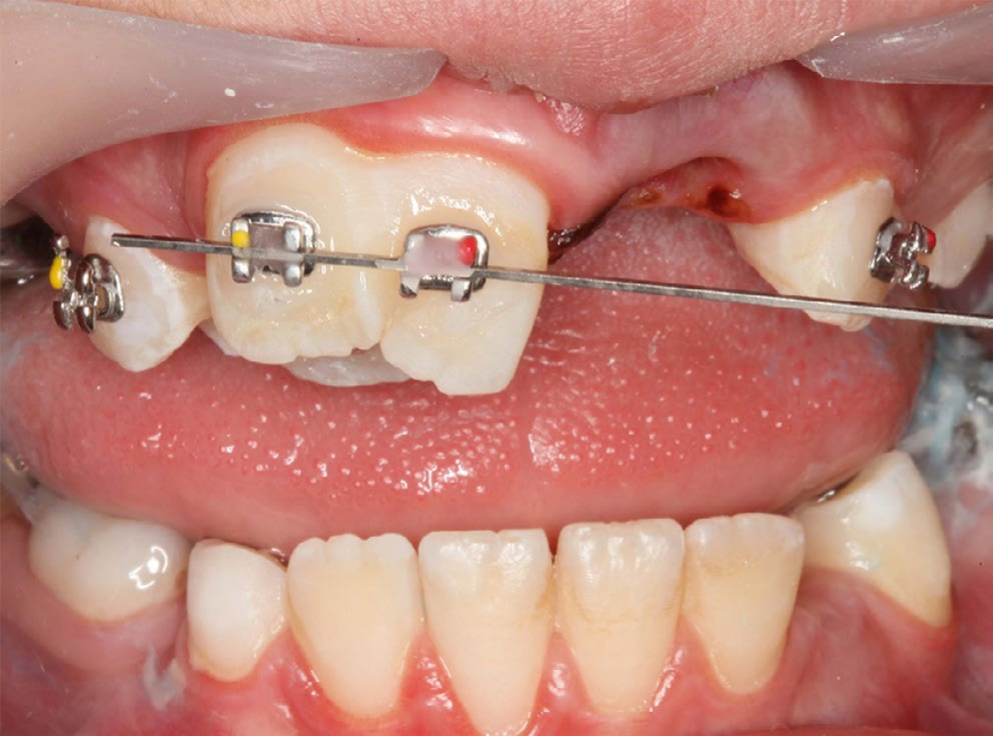
Bonding of two brackets to #11 with the help of a stiff wire (0.019 × 0.025‐in SS) to adjust both at a same vertical level

Two central incisor brackets with the help of a stiff wire, as shown in Figure 6, were bonded on the #11 at the same height to avoid unwanted forces among two parts of the fused tooth, and also to increase its mesiodistal control.

0.014‐in nickel titanium (Niti), 0.016‐in Niti, and 0.018‐in stainless steel (SS) were sequentially placed in 3 months. On 0.018‐in SS main archwire, two tieback loops mesial to the first molars were formed. On this 18 ss working wire, 150 gram mesialization force was delivered to the #11 by incorporation of a segment of Niti push coil at right and elastomeric chain at the left of fused incisor brackets.

**FIGURE 7:**
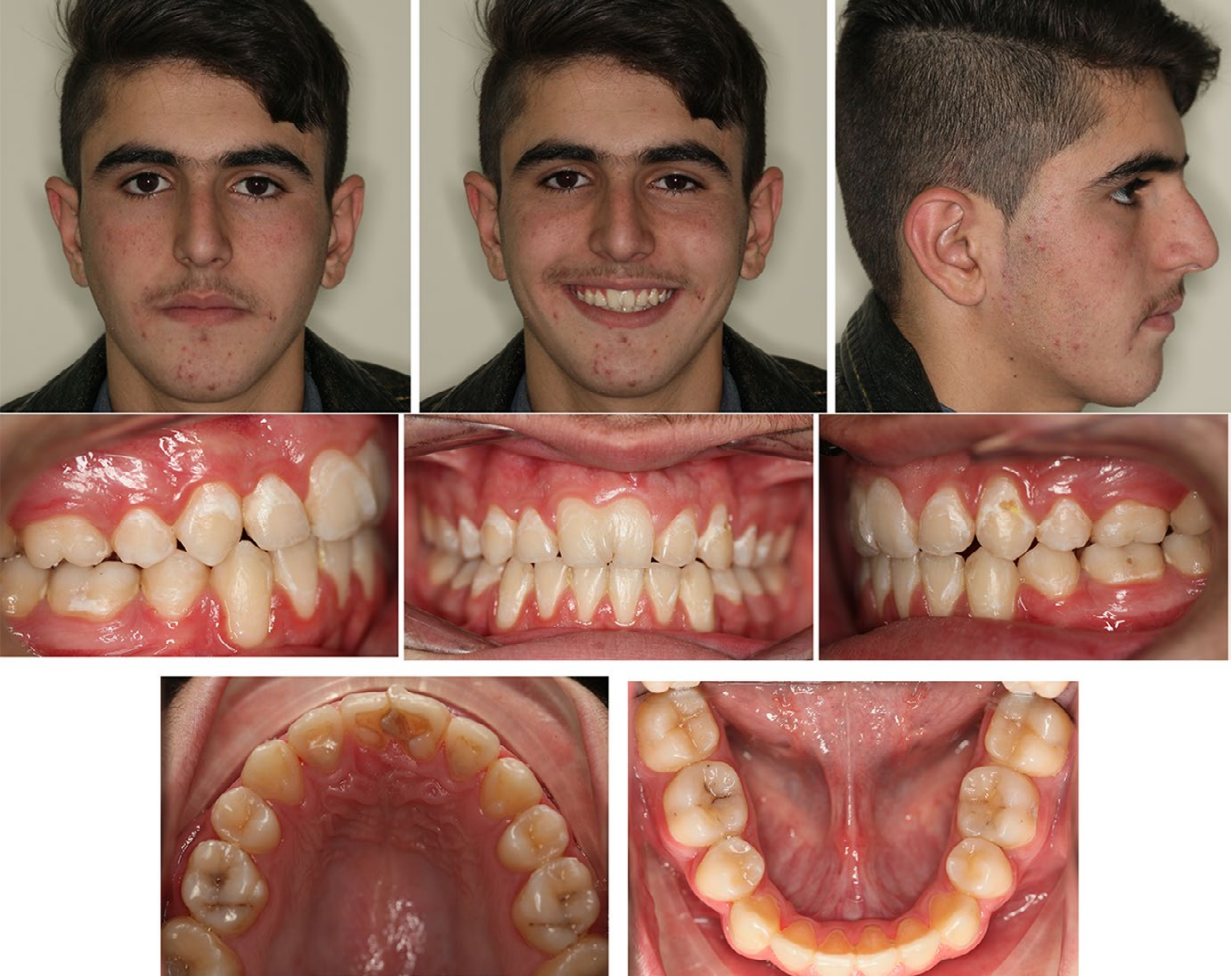
Posttreatment extraoral and intraoral photographs

**FIGURE 8:**
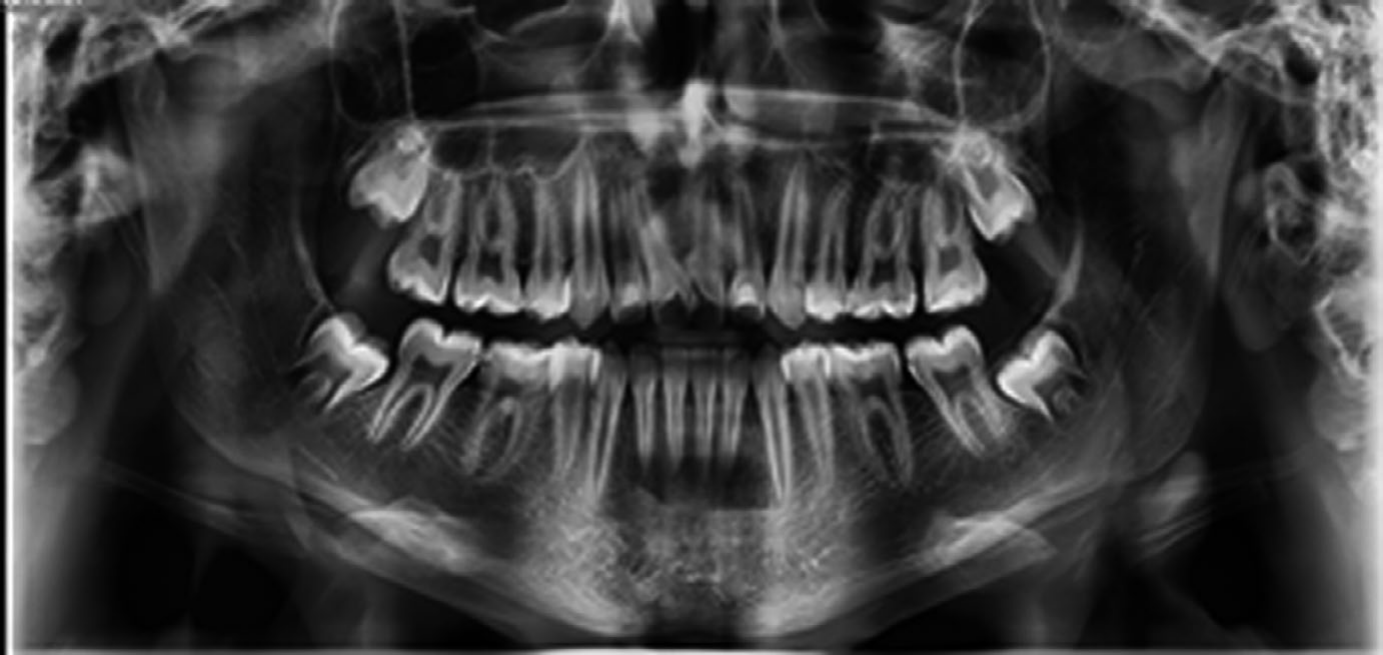
Posttreatment panoramic radiograph

**FIGURE 9:**
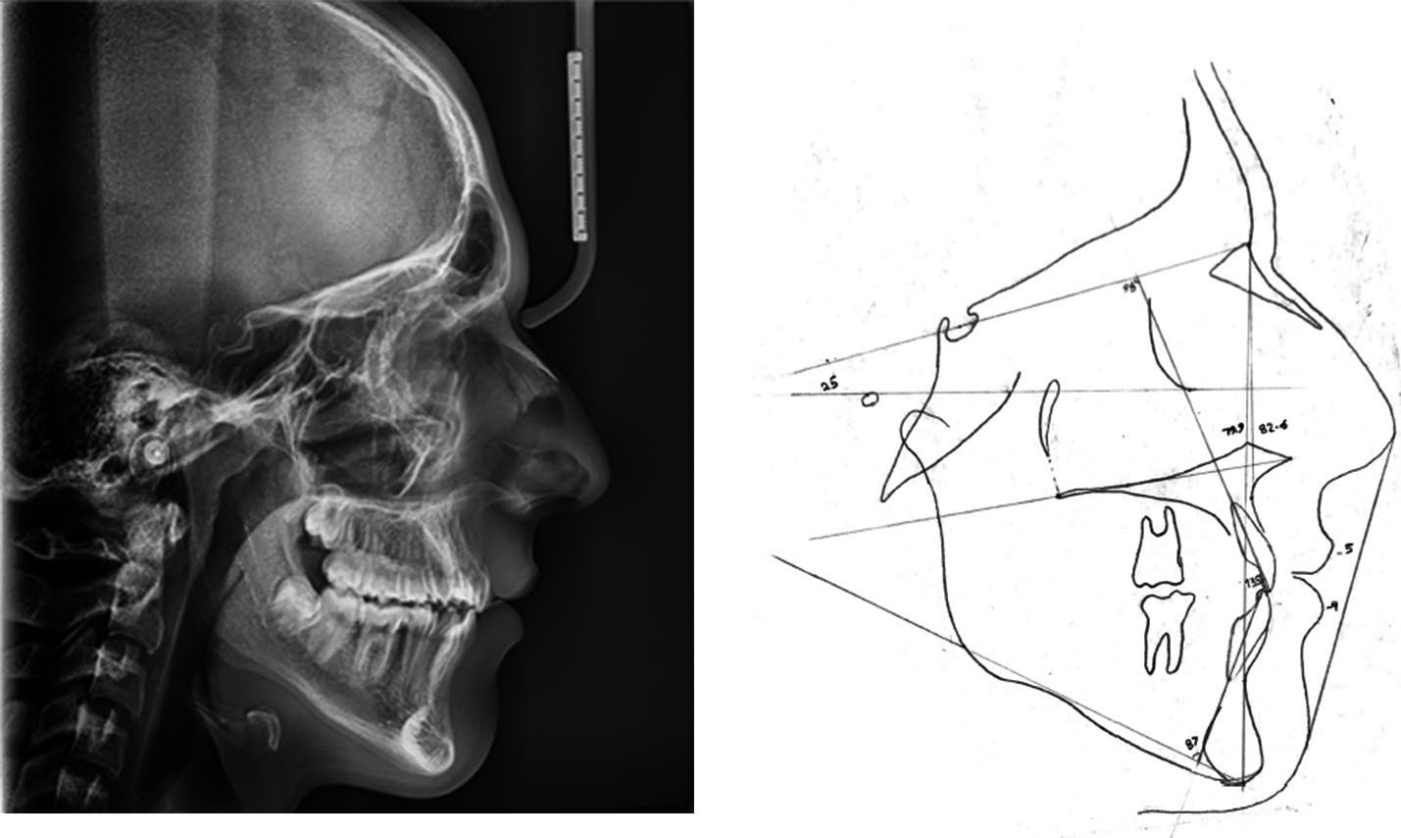
Posttreatment lateral cephalograms and tracings

**FIGURE 10:**
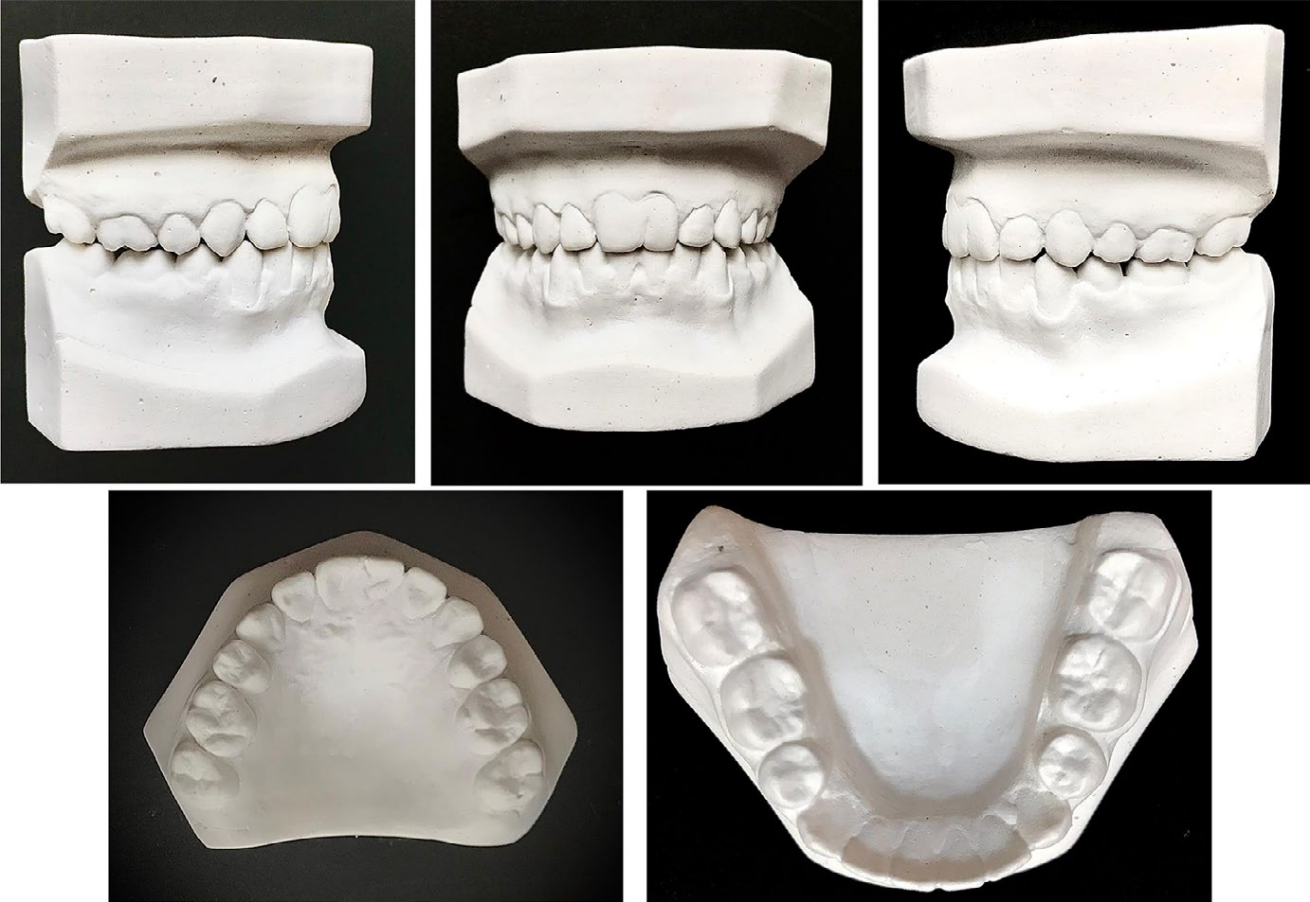
Posttreatment dental casts

**FIGURE 11:**
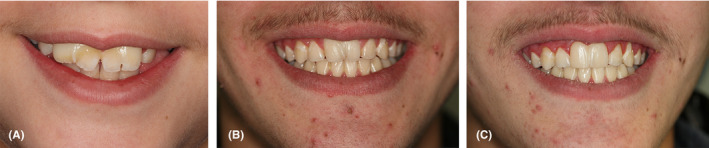
Smile close‐up photographs demonstrating the progress of esthetic smile, A, pretreatment smile photograph, B, post–orthodontic smile photograph, C, smile photograph after minor restorative corrections of #11

After midline correction in 10 months, sequential extraction of primary molars and first premolars, immediately after their eruption, was ordered. Leveling and alignment of the teeth were performed according to the standard protocols. Treatment was completed in 26 months (Figures 7, 8, 9, 10). Minor restorative treatment was done to slightly correct the form of the fused incisor (Figure 11). Hawley retainer for the upper and lower arch was provided for the patient, and the patient was instructed to wear it full time for 4 months and then night‐only for 1 year.

### 2.5 Treatment results

The facial profile remained unchanged after treatment (Table 1 and Figure 9). The patient's smile was balanced and pleasing. The final occlusion showed a class I occlusion with ideal overjet/overbite and coincident dental midlines. The maxillary right lateral incisor experienced palatal crown torque and labial root torque. In total, the incisor was moved across the MPS about 6 millimeters. The midline frenum was slightly stretched toward the left side but no major inflammation requiring periodontic procedures was observed. White spot lesions were observed particularly on the maxillary teeth. Based on the final radiographs, a proximation between the distal root of the fused incisor and the right lateral incisor was evident (All the other roots were parallel). That was inevitable because of the divergent roots of the fused incisor.

**TABLE 1:** Pretreatment and posttreatment cephalometric measurements

Analysis	Pretreatment	Posttreatment
Skeletal		
SNA (°)	82.9	82.6
SNB (°)	80	79.9
ANB (°)	2.9	2.7
SN‐GoGn (°)	38	36
Wits appraisal (mm)	3	3
Dental		
Interincisal angel (°)	136	135
IMPA (°)	88	87
U1‐SN (°)	98	98
Soft tissue		
Upper lip to S‐line (mm)	+4	+2
Upper lip to E‐line (mm)	−3	−5
Lower lip to E‐line (mm)	−1	−4

## 3 DISCUSSION

This case was a rare example of bilateral double teeth. Based on the literature, the prevalence of such a condition is 5 out of 100,000 in the human being population.^4^ Movement of birooted #11 through the MPS was successfully achieved. Indication of tooth movement through the MPS is closing a central incisor space that might be congenitally absent, severely malformed or hopeless to maintain. It aimed to avoid prosthetic treatment, reduce the number of extractions of healthy teeth, and help correct crowding and incisor proclination.^9^ Major considerations in moving teeth across the MPS are labial frenum inflammation and root resorption. Case studies reported that in growing patients that their suture is not yet ossified, moving incisors through the MPS would be less complicated, while in adult patients, root resorption was reported to be a serious accompanying effect of teeth crossing the ossified suture. In the present study, the patient was in the early mixed dentition at the beginning of the treatment and the speed of #11 mesialization was comparable to a normal‐sized tooth (0.98 mm per month). No dire root resorption was observed in this case, while in Kato's^9^ and Follin's^7^ studies, root resorption was considerable. Chiho Kato reported replacing a missing maxillary incisor with the contralateral one in a 26‐year‐old woman. In their case, while the maxillary left incisor crown crossed the midline and 8.7% of the apex remained on the same side, the root length shortened 3.3 mm.^9^

Bulut managed a case of fused maxillary central incisor by moving that across the MPS. In their case, no root resorption was observed; however, frenectomy had been performed, and also root canal therapy had to be done to prepare the tooth for casting restorations.^11^

Likewise in the present study, a mild stretch in the maxillary frenulum toward the movement of #11 (the left side) was observed. However, its inflammation was minor and under the control. Therefore, there was no need to perform frenectomy as advised by a periodontist.

A disadvantage of this treatment plan was the long period of treatment time (26 months). In spite of emphasis on oral hygiene, the patient had lost his cooperation which resulted in poor oral hygiene and extensive white spots. Dividing the treatment period into two separate phases would have prevented such complications.

From esthetic point of view, this case, although acceptable, it was not ideal. The upper and lower midlines were coincident. However, preservation of malformed incisor in front of the mouth was unattractive. A hemisection procedure to separate the teeth could be a solution. Since the #11 was asymptomatic and the patient was satisfied with the results, only a minor restorative correction was performed.

## 4 CONCLUSION

In complex cases of malformed teeth and severe crowding, it might be an appropriate option to move the tooth across the MPS in young patients.

## CONFLICT OF INTEREST

The authors declare that there is no conflict of interest in this study.

## AUTHOR CONTRIBUTIONS

S. Yassaei made the early diagnosis of the case and the main idea of the treatment plan also and had provision on all the stages of treatment. M. Sharifi contributed to providing the patients records, arranged the necessary dental procedure visits, and helped with drafting of the manuscript. Z. Ebrahiminik performed the clinical stages of patient treatment and visited the patient every month session and was a major contributor in drafting and submitting the manuscript.

## ETHICS APPROVAL AND CONSENT TO PARTICIPATE

The patient’s parents provided informed written consent prior to all interventions according to the requirements of the intuitional review board of the Faculty of Dentistry, Aja University of Medical Science.

## CONSENT FOR PUBLICATION

Informed written consent for publication was obtained from the patient’s parents

## Data Availability

The data that support the findings of this study are available from the corresponding author upon reasonable request.
